# A first digit theorem for powerful integer powers

**DOI:** 10.1186/s40064-015-1370-3

**Published:** 2015-10-06

**Authors:** Werner Hürlimann

**Affiliations:** Swiss Mathematical Society, University of Fribourg, 1700 Fribourg, Switzerland

**Keywords:** First digit, Powerful number, Asymptotic counting function, Probabilistic number theory, Mean absolute deviation, Probability weighted least squares, 11K36, 11N37, 11Y55, 62E20, 62F12

## Abstract

For any fixed power exponent, it is shown that the first digits of powerful integer powers follow a generalized Benford law (GBL) with size-dependent exponent that converges asymptotically to a GBL with the inverse double power exponent. In particular, asymptotically as the power goes to infinity these sequences obey Benford’s law. Moreover, the existence of a one-parametric size-dependent exponent function that converges to these GBL’s is established, and an optimal value that minimizes its deviation to two minimum estimators of the size-dependent exponent is determined. The latter is undertaken over the finite range of powerful integer powers less than $10^{s \cdot m} , \, m = 8, \ldots ,15$, where $s = 1,2,3,4,5$ is a fixed power exponent.

## Background

It is well-known that the first digits of many numerical data sets are not uniformly distributed. Newcomb (1881) and Benford (1938)) observed that the first digits of many series of real numbers obey *Benford’s law*

1 $$ P^{B} (d) = \log_{10} (1 + d) - \log_{10} (d),\quad d = 1,2, \ldots ,9. $$

The increasing knowledge about Benford’s law and its applications has been collected in two recent books by Berger and Hill (2015) and Miller (2015). In number theory, it is known that for any fixed power exponent $s \ge 1$, the first digits of some integer sequences, like integer powers and square-free integer powers, follow asymptotically a *Generalized Benford law* (GBL) with exponent $\alpha = s^{ - 1} \in (0,1]$ [see Hürlimann (2004), (2014a)] such that

2 $$ P_{\alpha }^{GB} (d) = \frac{{(1 + d)^{\alpha } - d^{\alpha } }}{{10^{\alpha } - 1}},\quad d = 1,2, \ldots ,9. $$

Clearly, the limiting case $\alpha \to 0$, respectively $\alpha = 1$, of (2) converges weakly to Benford’s law (1), respectively the uniform distribution. It is expected that many further integer power sequences follow a GBL with size-dependent parameter. However, if asymptotically such an exponent exists, it will not always be exactly $\alpha = s^{ - 1}$. Hürlimann (2014b) shows that the first digits of powers from perfect power numbers follow asymptotically a GBL with parameter $\alpha = (2s)^{ - 1}$.

Based on similar statistical analysis, the first digits of powerful integer powers are studied. For this, the GBL is fitted to samples of first digits using two goodness-of-fit measures, namely the MAD measure (mean absolute deviation) and the WLS measure (probability weighted least square or Chi square statistics). In “Size-dependent generalized Benford law for powerful integer powers”, the minimum MAD and WLS estimators of the GBL over finite ranges of powerful integer powers up to $10^{s \cdot m} , \, m \ge 8$, $s \ge 1$ a fixed power exponent, are determined. Calculations illustrate the convergence of the size-dependent GBL with minimum MAD and WLS estimators to the GBL with exponent $(2s)^{ - 1}$. Moreover, the existence of a one-parametric size-dependent exponent function that converges to these GBL’s is established, and an optimal value that minimizes its absolute deviation to the minimum MAD and WLS estimators is determined. A mathematical proof of the asymptotic convergence of the finite sequences to the GBL with exponent $(2s)^{ - 1}$ follows in “Asymptotic counting function for powerful integer powers”.

## Size-dependent generalized Benford law for powerful integer powers

A *powerful number* is a positive integer that is divisible by the square of each of its prime factors. It is of the form $n = a^{2} b^{3}$ for some natural numbers $a,\;b \ge 1$. To investigate the optimal fitting of the GBL to first digit sequences of powers from powerful numbers, it is necessary to specify goodness-of-fit (GoF) measures according to which optimality should hold. For this purpose, the following two GoF measures are used. Let $\{ x_{n} \} \subset [ 1 ,\infty ), \, n \ge 1$, be an integer sequence, and let $d_{n}$ be the (first) significant digit of $x_{n}$. The number of $x_{n}$’s, $n = 1, \ldots ,N$, with significant digit $d_{n} = d$ is denoted by $X_{N} (d)$. The *MAD measure* or *mean absolute deviation* measure for the GBL is defined to be

3 $$ MAD_{N} (\alpha ) = \frac{1}{9} \cdot \sum\limits_{d = 1}^{9} {\left| {P_{\alpha }^{GB} (d) - \tfrac{{X_{N} (d)}}{N}} \right|} . $$

This measure has been used to assess conformity to Benford’s law by Nigrini (2000) [see also Nigrini (2012), Table 7.1, p. 160]. The *WLS measure* for the GBL is defined by

4 $$ WLS_{N} (\alpha ) = \sum\limits_{d = 1}^{9} {\frac{{(P_{\alpha }^{GB} (d) - \tfrac{{X_{N} (d)}}{N})^{2} }}{{P_{\alpha }^{GB} (d)}}} . $$

Consider now the sequence of powerful integer powers $\{ n_{pf}^{s} \} , \, n_{pf}^{s} < 10^{s \cdot m}$, for a fixed power exponent $s = 1,2,3, \ldots$, and arbitrary powerful numbers $n_{pf}$ below $10^{m} , \, m \ge 8$. Denote by $I_{k}^{s} (d)$ the number of powerful integer powers below $10^{k} , \, k \ge 1$, with first digit $d$. This number is defined recursively by the relationship

5 $$ I_{k + 1}^{s} (d) = S(\sqrt[s]{{(d + 1) \cdot 10^{k} }}) - S(\sqrt[s]{{d \cdot 10^{k} }}) + I_{k}^{s} (d),\quad k = 1,2, \ldots , $$

where the counting function $S(n)$ is given by Golomb (1970) as

6 $$ S(n) = \sum\limits_{k = 1}^{{\left\lfloor {\sqrt[3]{n}} \right\rfloor }} {\mu^{2} (k) \cdot \left\lfloor {\sqrt {\tfrac{n}{{k^{3} }}} } \right\rfloor } , $$

where $\mu (k)$ is the Möbius function such that $\mu (k) = 0$ if $p^{2}$ divides $k$ and $\mu (k) = ( - 1)^{e}$ if $k$ is a square-free number with $e$ distinct prime factors, and $\left\lfloor \cdot \right\rfloor$ denotes the integer-part function. Recent algorithms to efficiently compute the Möbius function and the related counting function of square-free integers are contained in Pawlewicz (2011) and Auil (2013).

With $N = S(10^{m} )$ one has $X_{N} (d) = I_{s \cdot m}^{s} (d)$ in (3)–(4). A list of the $I_{s \cdot m}^{s} (d), \, m = 8, \ldots ,15$, $s = 1,2,3,4,5$, together with the sample size $N = S(10^{m} )$, is provided in Table 3 of the “Appendix”. Based on this, the so-called minimum MAD and minimum WLS estimators of the GBL are determined. Together with their GoF measures, these optimal estimators are reported in Table 1 below. Note that the minimum WLS is a critical point of the equation

7 $$ \begin{aligned} \frac{\partial }{\partial \alpha }WLS_{N} (\alpha ) = \frac{1}{N} \cdot \sum\limits_{d = 1}^{9} {\frac{{\partial P_{\alpha }^{GB} (d)}}{\partial \alpha } \cdot \frac{{P_{\alpha }^{GB} (d)^{2} - (\tfrac{{X_{N} (d)}}{N})^{2} }}{{P_{\alpha }^{GB} (d)^{2} }}} = 0, \hfill \\ \frac{{\partial P_{\alpha }^{GB} (d)}}{\partial \alpha } = \frac{{(1 + d)^{\alpha } \{ \ln (\tfrac{1 + d}{10})10^{\alpha } - \ln (1 + d)\} - d^{\alpha } \{ \ln (\tfrac{d}{10})10^{\alpha } - \ln (d)\} }}{{(10^{\alpha } - 1)^{2} }},\quad d = 1,2, \ldots ,9. \hfill \\ \end{aligned} $$

**Table 1:** GBL fit for first digit of powerful integer powers: MAD vs. WLS criterion

s = 1	Parameters		Δ to LL estimate		MAD GoF measures			WLS GoF measures		
m =	WLS	MAD	WLS	MAD	LL	WLS	MAD	LL	WLS	MAD
8	0.50630179	0.50602471	1.819	1.947	282.9	135.2	134.6	90.14	28.44	28.75
9	0.50398677	0.50439385	2.298	1.890	168.3	90.24	86.04	66.89	21.19	22.62
10	0.50258542	0.50277813	2.755	2.340	92.13	45.33	45.02	42.88	12.36	13.05
11	0.50170493	0.50180536	3.116	2.650	44.37	17.57	16.34	22.63	4.505	4.911
12	0.50116789	0.50121344	2.932	2.477	19.77	8.163	7.927	8.998	1.546	1.726
13	0.50080206	0.50083278	2.077	1.415	6.993	4.335	4.077	2.519	0.784	0.960
14	0.50054625	0.50054722	0.289	0.244	1.821	1.802	1.794	0.345	0.330	0.330
15	0.50037082	0.50036094	3.110	2.122	2.042	1.252	1.200	1.154	0.315	0.400

s = 2	Parameters		Δ to LL estimate		MAD GoF measures			WLS GoF measures		
m =	WLS	MAD	WLS	MAD	LL	WLS	MAD	LL	WLS	MAD
8	0.25428436	0.25324296	0.383	0.867	238.4	218.8	193.9	163.9	157.8	167.6
9	0.25428436	0.25267279	1.142	0.469	76.66	122.8	59.30	28.98	66.02	28.52
10	0.25166049	0.25155591	0.585	0.811	26.64	19.31	18.58	7.514	4.409	4.870
11	0.25096820	0.25089373	1.021	1.366	22.15	15.50	13.56	10.95	6.568	7.071
12	0.25062939	0.25059921	1.012	1.314	11.19	8.423	7.972	6.168	4.169	4.347
13	0.25042363	0.25041307	0.552	0.779	3.923	3.055	2.805	1.563	1.287	1.334
14	0.25028419	0.25028723	0.369	0.510	1.533	1.302	1.238	0.540	0.482	0.491
15	0.25018970	0.25019163	1.984	2.177	1.344	0.640	0.624	1.027	0.258	0.266

s = 3	Parameters		Δ to LL estimate		MAD GoF measures			WLS GoF measures		
m =	WLS	MAD	WLS	MAD	LL	WLS	MAD	LL	WLS	MAD
8	0.16900766	0.16723059	0.495	1.319	274.3	257.7	248.0	575.4	553.1	615.3
9	0.16801302	0.16762481	0.748	1.137	116.4	89.18	81.67	142.8	119.1	125.5
10	0.16769485	0.16753300	0.560	0.909	60.96	58.26	57.25	124.0	117.8	120.2
11	0.16729754	0.16730322	0.748	0.722	18.31	13.37	13.25	24.45	19.34	19.35
12	0.16710133	0.16708812	0.524	0.656	7.100	5.652	5.371	6.058	4.895	4.969
13	0.16695408	0.16695069	0.260	0.333	3.798	3.374	3.255	3.664	3.530	3.541
14	0.16685701	0.16685618	0.287	0.249	2.484	2.358	2.338	3.931	3.856	3.857
15	0.16679314	0.16679442	1.323	1.452	1.321	0.972	0.952	2.021	1.279	1.286

s = 4	Parameters		Δ to LL estimate		MAD GoF measures			WLS GoF measures		
m =	WLS	MAD	WLS	MAD	LL	WLS	MAD	LL	WLS	MAD
8	0.12774699	0.12760514	0.089	0.023	336.0	339.0	334.9	1704	1702	1703
9	0.12682400	0.12682674	0.253	0.256	108.7	100.3	100.2	495.4	489.5	489.6
10	0.12592594	0.12589200	0.086	0.160	38.75	37.58	36.59	149.2	148.9	149.1
11	0.12543815	0.12539409	0.724	0.928	21.98	16.83	15.38	52.95	42.62	43.45
12	0.12530767	0.12526505	0.576	1.002	7.884	6.275	5.841	18.01	14.97	16.63
13	0.12521961	0.12520948	0.108	0.326	4.885	4.688	4.291	20.84	20.79	20.99
14	0.12514734	0.12514391	0.428	0.269	1.315	1.279	1.181	4.693	4.331	4.381
15	0.12509739	0.12509771	1.246	1.278	0.940	0.587	0.579	3.039	1.619	1.620

s = 5	Parameters		Δ to LL estimate		MAD GoF measures			WLS GoF measures		
m =	WLS	MAD	WLS	MAD	LL	WLS	MAD	LL	WLS	MAD
8	0.10483305	0.10494392	1.295	1.346	551.6	498.4	496.2	8531	7818	7819
9	0.10110871	0.10166842	0.148	0.412	182.7	185.4	175.0	2055	2051	2113
10	0.10041545	0.10050001	0.770	0.588	39.74	30.51	28.02	211.5	157.1	160.2
11	0.10035943	0.10038750	0.538	0.407	21.67	19.45	18.85	124.1	111.8	112.5
12	0.10021129	0.10018888	0.809	1.033	10.05	8.099	7.558	60.76	47.82	48.81
13	0.10015612	0.10013482	0.508	0.967	5.373	4.752	4.238	35.74	33.38	35.31
14	0.10011058	0.10010322	0.004	0.337	1.790	1.792	1.653	9.827	9.827	10.32
15	0.10007403	0.10007147	0.609	0.353	0.794	0.713	0.655	4.477	3.745	3.874

For comparison, the MAD and WLS measures for the following size-dependent GBL exponent

8 $$ \alpha_{LL} (s \cdot m) = (2s)^{ - 1} \cdot \{ 1 + c \cdot 10^{ - a \cdot m} \} , $$

with $c = 1,\;a = 0.21119$, called LL estimator, are listed. This type of estimator is named in honour of Luque and Lacasa (2009) who introduced it in their GBL analysis for the prime number sequence. Through calculation one observes that the LL estimator minimizes the absolute deviations between the LL estimator and the MAD (resp. WLS) estimators over the finite ranges of powerful powers $[ 1 ,10^{s \cdot m} ] , \, m = 8, \ldots ,15, \, s = 1,2,3,4,5$. In fact, if one denotes the MAD and WLS estimators of the sequence $\{ n_{pf}^{s} \} , \, n_{pf}^{s} < 10^{s \cdot m}$, by $\alpha_{MAD} (s \cdot m)$ and $\alpha_{WLS} (s \cdot m)$, then one has uniformly over the considered finite ranges (consult the columns “Δ to LL estimate” in Table 1 in units of $\sqrt[3]{{10^{ - m} }}$)

9 $$ \begin{aligned} \left| {\alpha_{WLS} (s \cdot m) - \alpha_{LL} (s \cdot m)} \right| \le 3.116 \cdot \sqrt[3]{{10^{ - m} }}, \hfill \\ \left| {\alpha_{MAD} (s \cdot m) - \alpha_{LL} (s \cdot m)} \right| \le 2.650 \cdot \sqrt[3]{{10^{ - m} }}. \hfill \\ \end{aligned} $$

Table 1 displays exact results obtained on a computer with single precision, i.e., with 15 significant digits. The MAD (resp. WLS) measures are given in units of $10^{ - 6}$ (resp. $\sqrt[3]{{10^{ - (m + s + 12)} }}$). Taking into account the decreasing units, one observes that the optimal MAD and WLS measures decrease with increasing sample size.

## Asymptotic counting function for powerful integer powers

The following mimics Hürlimann (2014a), Section 3. It is well-known that a random process with uniform density $x^{ - 1}$ generates data that are Benford distributed. Similarly, a sequence of numbers generated by a power-law density $x^{ - \alpha } , \, \alpha \in [0,1]$, has a GBL first digit distribution $P_{1 - \alpha }^{GB} (d)$ with exponent $1 - \alpha$ [e.g. Pietronero et al. (2000), Eq. (3)]. From it, one derives a counting function $C(N)$ that yields the number of elements of that sequence in the interval $[ 1 ,N ]$. A local density of the form $x^{ - \alpha (x)}$, such that $C(N)\sim \int\nolimits_{2}^{N} {x^{ - \alpha (x)} dx}$, is usually not appropriate and must be modified as follows. The relation for powerful integer powers over an interval $[ 1 ,N^{s} ]$ that belongs to (8) is

10 $$ \alpha (N^{s} ) = \frac{2s - 1 - \alpha (N)}{2s},\quad \alpha (N) = c \cdot N^{ - a} ,\quad a = 0.21119,\quad c \ge 1, $$

Denote by $Q_{s} (N^{s} )$ the counting function for powerful integer powers in the interval $[ 1 ,N^{s} ]$. Instead of $\int\nolimits_{2}^{{N^{s} }} {x^{{ - \alpha (N^{s} )}} dx}$ define

11 $$ Q_{s} (N^{s} ) = q \cdot (2s)^{ - 1} \cdot \int\limits_{2}^{{N^{s} }} {x^{{ - \alpha (N^{s} )}} dx} ,\quad q = \frac{{\zeta (\tfrac{3}{2})}}{\zeta (3)} = 2.17325, $$

with $\zeta ( \cdot )$ the Riemann zeta function. In this expression, the integral pre-factor is chosen to fulfill the asymptotic limiting value for the powerful number counting function, that is (note that $n_{pf}^{s} < N^{s}$ if, and only if, one has $n_{pf} < N$)

12 $$ \mathop {\lim }\limits_{N \to \infty } \frac{{Q_{s} (N^{s} )}}{\sqrt N } = q. $$

In fact, two analytical bounds on $S(N)$ are known, namely

13 $$ q \cdot \sqrt N - 3 \cdot \sqrt[3]{N} \le S(N) \le q \cdot \sqrt N ,{\text{ and}} $$

14 $$ q \cdot \sqrt N - 1.83522 \cdot \sqrt[3]{N} \le S(N) \le q \cdot \sqrt N - 1.207684 \cdot \sqrt[3]{N},\quad N \ge 961. $$

The first one is classical and proved in Golomb (1970). The second improved estimate is due to Mincu and Panaitopol (2009), Theorem 1. However, it suffices to use the simple estimate (12), which is obtained as follows. From (11) one gets for arbitrary $s = 1,2, \ldots$, taking into account (10), the equivalent asymptotic formula

15 $$\begin{aligned}
Q_{s}(N^{s})&\sim \frac{q}{2s \cdot (1 - \alpha (N^{s}))} \cdot N^{s
\cdot (1 - \alpha (N^{s} ))} Q_{s} (N^{s}) \\
&= \frac{q}{1 + \alpha (N)} \cdot N^{\tfrac{1}{2}(1 + \alpha (N))} =
q \cdot {\sqrt{N}} \cdot \frac{N^{a}}{N^{a}+ c} \cdot \exp \left(
{\tfrac{1}{2}c\frac{\ln (N)}{N^{a}}} \right)
\end{aligned}$$

which is independent of $s$ and simply denoted by $Q(N)$. The equality $Q_{s} (N^{s} ) = Q(N)$ reflects the fact that there are as many powerful integer powers in $[ 1 ,N^{s} ]$ as there are powerful numbers in $[ 1 ,N ]$. Now, what is a good value of $c \in [ 1 ,\infty )$? Clearly, the factor

16 $$ f_{N} (c) = \frac{{N^{a} }}{{N^{a} + c}} \cdot \exp \left( {\tfrac{1}{2}c\frac{\ln (N)}{{N^{a} }}} \right) $$

converges from above to 1 as $N \to \infty$ for any fixed $c$. Its derivative with respect to $c$ satisfies the property $\tfrac{\partial }{\partial c}f_{N} (c) > 0, \, \forall \;c \in [ 1 ,\infty ) , \, \forall \;N \ge 4$. Since $f_{N} (c)$ increases in $c$ and decreases in $N$, one has the following max–min property of (16) at $c = 1$:

17 $$ \mathop {\hbox{max} }\limits_{{N \ge 10^{16} }} \left \{ \mathop {\hbox{min} }\limits_{c \in [ 1 ,\infty ) } f_{N} (c) \right \} = f_{{10^{16} }} (1) = 1.0073. $$

Therefore, the size-dependent exponent (10) with $c = 1$ not only minimizes the absolute deviations between the LL estimator and the MAD (resp. WLS) estimators over the finite ranges of powerful powers $[ 1 ,10^{s \cdot m} ] , \, m = 8, \ldots ,15, \, s = 1,2,3,4,5$, as shown in “Size-dependent generalized Benford law for powerful integer powers”, but it turns out to be uniformly best with maximum error less than $7.3 \cdot 10^{ - 3}$ against the asymptotic estimate, at least if $N \ge 10^{16}$. Moreover, the following limiting asymptotic result has been shown.

### First digit theorem for powerful integer powers (*GBL for powerful integer powers*)

The asymptotic distribution of the first digit of powerful integer power sequences $n_{pf}^{s} < 10^{s \cdot m} , \, m \ge 8$, for fixed $s = 1,2,3, \ldots$, as $m \to \infty$, is given by

18 $$ \begin{aligned} \begin{array}{l} \mathop {\lim }\limits_{m \to \infty } \frac{{I_{s \cdot m}^{s} (d)}}{{S(10^{m} )}} = \mathop {\lim }\limits_{m \to \infty } P_{\alpha (s \cdot m)}^{GB} (d) = P_{{(2s)^{ - 1} }}^{GB} (d),\quad d = 1, \ldots ,9, \hfill \\ \alpha (s \cdot m) = \frac{1}{2s}\left( {1 + \frac{1}{{10^{a \cdot m} }}} \right),\quad a = 0.21119. \hfill \\ \end{array} \end{aligned} $$

The next Table 2 compares the new counting function $Q(N)/q\sqrt N$ with the lower bound for $S(N)/q\sqrt N$ in (14), as well as the ratios of these with the asymptotic value $q \cdot \sqrt N$. Clearly, the new counting function is an approximation to the latter from above.

**Table 2:** Comparison of powerful number counting functions for $N = 10^{m}$

m	*S* (*N)* lower bound (14)	*Q* (*N)*	*S* (*N)*/*q √N*	*Q* (*N)*/*q √N*
8	20,880	25,708	0.960773	1.182929
9	66,888	77,311	0.973282	1.124946
10	213,371	235,726	0.981806	1.08467
11	678,723	726,421	0.987604	1.057009
12	2,154,897	2,256,191	0.991555	1.038165
13	6,832,881	7,047,093	0.994247	1.025417
14	21,647,316	22,098,596	0.99608	1.016846
15	68,540,677	69,488,109	0.99733	1.011116
16	216,929,613	218,912,528	0.998181	1.007305
17	686,390,158	690,528,822	0.998761	1.004783
18	2,171,414,780	2,180,031,831	0.999156	1.003121
19	6,868,466,063	6,886,369,416	0.999425	1.00203
20	21,723,981,663	21,761,110,713	0.999608	1.001316
21	68,705,847,049	68,782,727,400	0.999733	1.000852
22	217,285,461,383	217,444,443,741	0.999818	1.00055
23	687,156,809,129	687,485,219,306	0.999876	1.000354
24	2,173,066,478,000	2,173,744,295,846	0.999916	1.000227
25	6,872,024,538,797	6,873,422,599,112	0.999942	1.000146

## Conclusions

The first digits of some integer sequences like integer powers and square-free integer powers to a fixed power exponent $s \ge 1$, follow a generalized Benford law with size-dependent exponent that converges asymptotically to a GBL with the inverse power exponent $s^{ - 1}$. In contrast to this, there exist integer sequences, for which such power sequences behave like a GBL with parameter different from $s^{ - 1}$. The analysed powerful integer power sequences follow a GBL with parameter $(2s)^{ - 1}$ and are in this respect similar to powers from perfect power numbers studied previously in Hürlimann (2014b). Moreover, all these power sequences typically are not exactly Benford distributed. Departures from Benford’s law occur quite frequently within mathematics and in almost all related scientific disciplines, and must be analysed along the line of appropriate probabilistic models. The companion papers Hürlimann (2015a, b) introduce and discuss some possibilities for a scientific wider use.

## Appendix

Based on the recursive relation (5)–(6), the calculation of $I_{s \cdot m}^{s} (d),\quad m = 8, \ldots ,15$, is straightforward, at least if a table of the Möbius function is available [e.g. sequence A001694 in OEIS founded by Sloane (1964)]. These numbers are listed in Table 3. The entry $s \to \infty$ corresponds to the limiting Benford law as the power goes to infinity.

**Table 3:** First digit distribution of powerful integer powers up to $10^{s \cdot m} , \, m = 4, \ldots ,10$

s = 1/1st digit	21,044	67,231	214,122	680,330	2,158,391	6,840,384	21,663,503	68,575,557
1	4007	12,822	40,904	130,091	412,967	1,309,267	4,147,585	13,131,523
2	3083	9871	31,450	99,940	317,119	1,005,145	3,183,613	10,078,415
3	2610	8331	26,524	84,296	267,441	847,620	2,684,438	8,497,665
4	2300	7347	23,395	74,293	235,677	746,893	2,365,308	7,487,199
5	2073	6636	21,136	67,171	213,092	675,309	2,138,598	6,769,360
6	1913	6110	19,454	61,808	196,043	621,153	1,966,875	6,225,554
7	1786	5696	18,119	57,541	182,478	578,178	1,830,822	5,794,823
8	1682	5363	17,044	54,078	171,442	543,121	1,719,686	5,442,855
9	1590	5055	16,096	51,112	162,132	513,698	1,626,578	5,148,163

s = 2/1st digit	21,044	67,231	214,122	680,330	2,158,391	6,840,384	21,663,503	68,575,557
1	5101	16,305	51,985	165,261	524,441	1,662,341	5,265,235	16,668,479
2	3423	10,953	34,878	110,838	351,732	1,114,820	3,530,987	11,177,711
3	2653	8468	26,991	85,800	272,191	862,616	2,731,775	8,647,291
4	2194	7011	22,334	70,939	225,030	713,170	2,258,528	7,149,260
5	1882	6029	19,196	60,981	193,455	613,024	1,941,382	6,145,104
6	1662	5320	16,935	53,789	170,584	540,582	1,711,903	5,418,705
7	1506	4784	15,209	48,289	153,178	485,450	1,537,240	4,865,873
8	1360	4348	13,848	43,989	139,490	441,889	1,399,282	4,429,024
9	1263	4013	12,746	40,444	128,290	406,492	1,287,171	4,074,110

s = 3/1st digit	21,044	67,231	214,122	680,330	2,158,391	6,840,384	21,663,503	68,575,557
1	5506	17,585	56,011	178,000	564,823	1,790,255	5,670,207	17,949,938
2	3521	11,272	35,904	114,105	362,030	1,147,413	3,633,936	11,503,374
3	2646	8467	26,989	85,776	272,126	862,442	2,731,429	8,646,513
4	2148	6855	21,840	69,434	220,301	698,158	2,210,950	6,998,516
5	1818	5817	18,502	58,717	186,183	590,041	1,868,635	5,914,789
6	1583	5046	16,076	51,051	161,930	513,041	1,624,701	5,142,951
7	1403	4473	14,233	45,264	143,639	455,234	1,441,644	4,563,270
8	1254	4032	12,857	40,802	129,418	410,055	1,298,522	4,110,259
9	1164	3683	11,709	37,180	117,940	373,744	1,183,478	3,745,946

s = 4/1st digit	21,044	67,231	214,122	680,330	2,158,391	6,840,384	21,663,503	68,575,557
1	5698	18,216	58,062	184,559	585,590	1,855,943	5,878,103	18,607,799
2	3577	11,427	36,392	115,638	366,864	1,162,715	3,682,401	11,656,783
3	2656	8466	26,970	85,665	271,811	861,509	2,728,356	8,636,596
4	2110	6777	21,591	68,632	217,705	689,893	2,184,938	6,916,359
5	1790	5702	18,115	57,504	182,437	578,178	1,831,099	5,796,212
6	1528	4891	15,615	49,655	157,550	499,308	1,581,133	5,004,805
7	1350	4335	13,793	43,814	138,938	440,298	1,394,424	4,413,972
8	1226	3888	12,354	39,236	124,518	394,542	1,249,524	3,955,235
9	1109	3529	11,230	35,627	112,978	357,998	1,133,525	3,587,796

s = 5/1st digit	21,044	67,231	214,122	680,330	2,158,391	6,840,384	21,663,503	68,575,557
1	5809	18,610	59,329	188,524	598,205	1,895,921	6,004,544	19,007,822
2	3609	11,527	36,670	116,505	369,658	1,171,550	3,710,470	11,745,615
3	2640	8451	26,948	85,615	271,535	860,564	2,725,421	8,627,359
4	2126	6756	21,449	68,139	216,096	684,815	2,168,758	6,865,158
5	1738	5591	17,871	56,780	180,199	571,066	1,808,411	5,724,220
6	1509	4821	15,356	48,817	154,913	490,922	1,554,819	4,921,783
7	1323	4248	13,510	42,921	136,137	431,469	1,366,413	4,325,270
8	1202	3805	12,076	38,352	121,654	385,457	1,220,575	3,863,552
9	1088	3422	10,913	34,677	109,994	348,620	1,104,092	3,494,778

s → ∞/1st digit	21,044	67,231	214,122	680,330	2,158,391	6,840,384	21,663,503	68,575,557
1	6335	20,239	64,457	204,800	649,740	2,059,161	6,521,364	20,643,300
2	3706	11,839	37,705	119,800	380,074	1,204,532	3,814,754	12,075,556
3	2629	8400	26,752	85,000	269,667	854,629	2,706,611	8,567,743
4	2039	6515	20,751	65,931	209,170	662,902	2,099,410	6,645,658
5	1666	5323	16,954	53,869	170,904	541,630	1,715,343	5,429,898
6	1409	4501	14,335	45,546	144,497	457,942	1,450,302	4,590,913
7	1220	3899	12,417	39,454	125,169	396,687	1,256,309	3,976,830
8	1076	3439	10,953	34,801	110,407	349,903	1,108,143	3,507,813
9	963	3076	9798	31,130	98,763	312,999	991,268	3,137,845

## References

[CR1] Auil F (2013). An algorithm to generate square-free numbers and to compute the Möbius function. J Number Theory.

[CR2] Benford F (1938). The law of anomalous numbers. Proc Am Phil Soc.

[CR3] Berger A, Hill TP (2015). An introduction to Benford’s law.

[CR4] Golomb SW (1970). Powerful numbers. Am Math Monthly.

[CR5] Hürlimann W (2004). Integer powers and Benford’s law. Int J Pure Appl Math.

[CR6] Hürlimann W (2014). A first digit theorem for square-free integer powers. Pure Math Sci.

[CR7] Hürlimann W (2014). A first digit theorem for powers of perfect powers. Commun Math Appl.

[CR8] Hürlimann W (2015). On the uniform random upper bound family of first significant digit distributions. J Inf.

[CR9] Hürlimann W (2015). Benford’s law in scientific research. Int J Sci Eng Res.

[CR10] Luque B, Lacasa L (2009). The first digit frequencies of prime numbers and Riemann zeta zeros. Proc Royal Soc A.

[CR11] Miller SJ (2015). Benford’s law: theory and applications.

[CR12] Mincu G, Panaitopol L (2009) More about powerful numbers. Bull Math Soc Sci Math Roumanie 52(100):451–460 **(No. 4)**

[CR13] Newcomb S (1881). Note on the frequency of use of the different digits in natural numbers. Am J Math.

[CR14] Nigrini MJ (2000). Digital analysis using Benford’s law: test statistics for auditors.

[CR15] Nigrini MJ (2012). Benford’s law. Applications for forensic accounting, auditing, and fraud detection.

[CR16] Pawlewicz J (2011) Counting square-free numbers. arXiv:1107.4890v1 [math.NT]

[CR17] Pietronero L, Tossati E, Tossati V, Vespignani A (2000). Explaining the uneven distribution of numbers in nature: the laws of Benford and Zipf. Physica A.

[CR18] Sloane NJA (1964) The on-line encyclopedia of integer sequences. https://oeis.org/

